# The time of recognition: a time-critical theory of social misrecognition in welfare societies

**DOI:** 10.3389/fsoc.2025.1601359

**Published:** 2025-08-14

**Authors:** Gottfried Schweiger

**Affiliations:** Centre for Ethics and Poverty Research, University of Salzburg, Salzburg, Austria

**Keywords:** recognition, time, poverty, precarization, life course, temporal regime, social critique

## Abstract

This paper analyzes recognition as a temporal phenomenon. It seeks to better understand how the temporality of recognition—that is, when it is demanded, received, and given, and for how long—is structured in modern societies, and what the consequences are when these temporal structures erode or collapse. To this end, the paper focuses on two social processes: poverty and the precarization of work. It argues that both are better understood through the lens of the temporal dimension of recognition—or more precisely, the temporal dimension of non-recognition and misrecognition. This perspective reveals that poverty and precarization are temporally extended forms of non-recognition. It becomes clear that the timing of these experiences within a person’s life course, their duration, and the temporal regimes established by welfare states all play a crucial role. These regimes often exclude and disadvantage those affected, reinforcing their marginalization over time.

## 1 Introduction

The theory of recognition has, over the past decades, developed into a significant paradigm within social philosophy and social theory (Honneth, 1996; O’Neill and Smith, 2012; Deranty et al., 2007). Especially through the work of Axel Honneth, it has become a tool not only for reconstructing social relations but also for critically evaluating them. At its core is the assumption that successful recognition is a prerequisite for personal integrity, social participation, and a coherent relationship to oneself, while forms of non-recognition or misrecognition lead to psychological harm, social conflict, and societal pathologies. The three forms of recognition that Honneth distinguishes (Honneth, 1996)—love, rights or respect, and social esteem—are not merely normative ideals but institutionally mediated structures that shape both social coexistence and individual life trajectories. Poverty, from this perspective, does not merely appear as a lack of economic resources but as an expression of a deeper problem: a structural lack of recognition (Schweiger, 2020b, 2024). Those living in poverty not only experience social exclusion but often also a systematic form of invisibility, degradation, and exclusion from the social relations in which recognition is mediated. Poverty is therefore not only economically, but also socially and normatively relevant. It points to damaged relations of recognition, to missing belonging, and to the failure of society to secure a place for all its members within the symbolic order of social esteem. It can be shown that poverty in modern capitalist welfare states is also temporally structured (Dewilde, 2003; Hübgen, 2018; Harriss, 2009). Recognition is not something momentary or one-time, but unfolds over time, in concrete biographical trajectories and institutional processes (Schweiger, 2020a, 2022). People experience recognition—or its absence—not only in the moment, but over time, in cumulative experiences, in disappointments and long-term expectations that remain unfulfilled. The experience of poverty often means that recognition comes too late, does not last long enough, or is permanently withheld. It is these processes of disappointed expectation, ongoing denial, and lack of predictability that render poverty intelligible as a temporal phenomenon. In modern societies, the life course is heavily prestructured by institutions (Kohli, 2007). Education, gainful employment, and retirement are not only social facts but normative orders that present a model of a successful life. Those who deviate from this—through unemployment, precarious employment, or long-term poverty—fall not only behind materially but also into a zone of social non-recognition. Social esteem is built up over time, for example through work biographies or family responsibilities. Those who do not meet these expectations are often not merely overlooked but actively devalued. From this perspective, poverty means not finding a place within the established temporal regimes of recognition—or being excluded from them.

The aim of this work is to systematically analyze the connection between poverty and recognition by adopting a social-theoretical perspective that considers both the normative structure of recognition relations and their temporal dimension. The theory of recognition is not understood here as a closed system but as an open research program capable of capturing empirical phenomena such as poverty, precarity, or social exclusion in their structural, normative, and temporal interconnections. The paper is divided into five sections: following the introduction, the fundamental features of the theory of recognition and its temporal structure are elaborated. The third section provides an analysis of poverty, precarious employment, and the significance of the life course in the conpaper of recognition and misrecognition. The fourth section addresses a critique of current societal time regimes that promise recognition but fail to deliver it. Finally, the findings are brought together and a perspective on further research questions is outlined.

## 2 Fundamental features of the theory of recognition and its temporal dimension

The theory of recognition was significantly developed by Axel Honneth as a normatively oriented social philosophy in which the moral prerequisites of successful self-relations are linked to a diagnosis of societal pathologies (Honneth, 2014; Honneth, 1996; Petherbridge, 2013). At its core, it aims to reconstruct the conditions under which people can experience themselves as autonomous, equal, and valuable subjects. Honneth draws on social-psychological, philosophical, and social-theoretical sources, particularly George H. Mead, Donald Winnicott, and Hegel’s early work. From this perspective, recognition is not a voluntary act of attention but a necessary condition for the development of a stable relation to oneself. People depend on recognition in order to develop self-confidence, self-respect, and self-esteem—each mediated through specific social relationships. Honneth distinguishes three forms of recognition: love, respect, and social esteem. Love refers to emotional attention in primary relationships such as family, friendship, and partnership, forming the foundation for the development of self-confidence. Respect refers to legal equality and recognition as an autonomous subject with rights—a form of recognition realized in the institutions of law, politics, and the public sphere. Social esteem refers to the recognition of individual abilities, achievements, and contributions to the community, particularly in the sphere of gainful employment. Each of these forms is not only normatively significant but institutionally embedded. They are anchored in social practices, expectations, and structures that are historically evolved and in principle changeable.

Recognition is thus not limited to intersubjective encounters but permeates the social order as a whole. This perspective makes it possible to understand social institutions not merely as functional but as normative orders that enable or withhold recognition. Societies can thus be reconstructed as institutionalized orders of recognition. Within them, it is decided who is deemed worthy of recognition, who receives which rights, and whose achievements are met with social esteem. This institutional dimension is central to understanding social inequality, exclusion, and conflict. Social struggles, in this framework, are not merely conflicts over resources but also over recognition—that is, over visibility, value, and belonging (Voswinkel, 2001, 2012b; Honneth, 2012; Tully, 2000). Less emphasized, but theoretically central, is the temporal structure of recognition. Recognition is not a singular event but unfolds over time. It possesses duration, temporal reach, and social rhythm. Those who experience recognition do so in specific phases of life, at certain times, and often in response to earlier expectations or actions. Recognition in childhood—for instance, through parental care—lays the foundation for later self-relations. In adulthood, respect and social esteem gain importance, mediated by legal equality and societal participation, especially through employment. These forms of recognition, however, are not evenly distributed across life but are subject to biographical orders, institutional expectations, and normative time regimes.

The biographical impact of these temporal recognition structures can be illustrated through empirical findings from qualitative studies on long-term unemployment, which demonstrate how the erosion of institutionalized recognition promises manifests not only in employment discontinuity but also in the systematic devaluation of qualifications acquired over decades (Chassé et al., 2011). Research by Daly and Kelly (2015) on families living in poverty reveals how the relationship to time becomes fundamentally altered in precarious living conditions—shifting from bourgeois conceptions of plannable, linearly progressive biographies toward a mode of permanent crisis management in which the present becomes mere survival and the future loses its orienting force. What emerges from these studies is a pattern whereby temporal expectations that once structured life courses—such as the assumption that continuous work effort would lead to security and recognition—transform into sources of chronic disappointment, thereby undermining both self-perception and future expectations in ways that extend far beyond immediate material deprivation (Underlid, 2005). The significance of these temporal disruptions lies not merely in their immediate effects but in their cumulative biographical sedimentation, which creates what might be termed “temporal injuries” that persist across life phases and contribute to the reproduction of social marginalization through damaged relationships to both past achievements and future possibilities.

Drawing upon the theoretical foundations of recognition theory, a systematic distinction between non-recognition and misrecognition emerges as central to understanding temporal recognition dynamics—a differentiation that, building on Stephan Voswinkel’s early critique of Honneth, rests fundamentally on the distinction between passive and active forms of recognition denial (Voswinkel, 2001, 2012a). While non-recognition can be understood primarily as a passive form of ignorance—whereby recognition claims are simply overlooked, neglected, or systematically disregarded, manifesting itself in the societal invisibility of certain groups, in institutional indifference toward precarious living conditions, or in the structural exclusion of care work from public discourse—misrecognition, by contrast, designates active forms of degradation, discreditation, or normative distortion that are characterized through targeted actions, explicit devaluation, or deliberate misinterpretation of persons and their contributions. It is particularly noteworthy that this differentiation, while analytically useful, should not obscure the fact that both forms can be equally harmful and that the boundaries between passive ignorance and active harm often prove fluid—since ignoring someone frequently involves deliberate action to disregard their recognition claims, and since there exist numerous borderline cases. The temporal dimension of this distinction proves analytically fruitful precisely because both forms of recognition denial exhibit different temporal structures and unfold different biographical effects: non-recognition frequently manifests as chronic absence of attention, as systematic overlooking over extended periods, or as institutional neglect that settles into what Schweiger (2020a) identifies as “enduring denial”—that form of persistent recognition refusal that constitutes a central experience for people in poverty—while misrecognition often occurs episodically but, through its intensity and explicit nature, can cause profound and lasting psychological damage that remains effective as “long term suffering” beyond immediate recognition experiences. In modern welfare societies, this dynamic becomes particularly evident in the treatment of poverty and precarious employment relationships, where people are both systematically overlooked—for instance, when their work in care sectors receives no societal recognition, or when their needs find no consideration in political decision-making processes—and actively stigmatized and discredited as “failures” or “parasites,” whereby this dual structure of non-recognition and misrecognition sediments biographically and leads to those “disappointed expectations” that Schweiger describes as characteristic of the “life course of poverty” (Schweiger, 2020b; Schweiger, 2020a). The temporal analysis of these phenomena clarifies that both passive denial and active distortion of recognition must be understood not as isolated events but as structural processes that accumulate across life courses, thereby establishing specific temporal regimes of exclusion in which people are not only marginalized in the present but also deprived of future opportunities and damaged in their biographical continuity—a process that becomes particularly visible in the systematic “lack of predictability” that characterizes precarious life conditions, where people cannot develop stable expectations about whether they will be treated with respect or subjected to humiliation.

In the institutional structure of modern societies, this is reflected in the form of the standardized life course (Kohli, 2007, 2023; Scherger, 2024). The life course is more than a descriptive sequence of phases of education, work, and retirement; it is a normative framework that dictates when certain forms of recognition are expected, granted, or denied. The idea of an “institutionalized life course” describes the temporal organization of societal relations of recognition: childhood as a phase of care and learning, working life as a phase of performance and social productivity, retirement as a phase of rest and deferred reward. This structure is not neutral but normatively charged—those who deviate from it are often not only materially disadvantaged but also symbolically devalued.

The temporal structure of recognition can be analytically differentiated along two dimensions (Schweiger, 2022): duration and timing. Duration concerns the question of how long recognition is granted or denied, whether it is experienced continuously or remains episodic, interrupted, fragile. Timing concerns the moment at which recognition occurs—whether it comes too early, too late, or not at all. Both dimensions are normatively significant. Recognition that briefly flares up and quickly fades cannot fulfill its stabilizing function. Recognition that is delayed or premature misses social expectations and generates irritation, disappointment, or frustration. Recognition is thus a relation in time, whose success or failure crucially depends on its temporal structure. The experience of non-recognition, from this perspective, is also not merely momentary but can be understood as a process—as a chronic form of misrecognition, as a permanently denied sense of belonging, as a biographically entrenched feeling of insignificance and worthlessness. Non-recognition can accumulate, intensify, become habitual—particularly when it is systematically anchored in institutional practices. Especially in complex societies, where life is structured by long-term planning, expectations, and norms, the temporal dimensions of recognition are central to understanding exclusion and inequality.

This becomes particularly clear in the realm of employment. In modern societies, work is not only a means of securing material existence but also a central medium of social integration and esteem (Holtgrewe et al., 2001; Honneth, 2010; Voswinkel, 2012b). Gainful employment mediates recognition by making achievements visible, enabling social contributions, and generating income, which in turn is read as an expression of social appreciation. At the same time, employment itself is embedded in temporal structures—for example, in the model of standard employment, which promises stability, continuity, and predictability. Those who are permanently unemployed or only engage in precarious employment experience not only economic insecurity but also a long-term erosion of social esteem. Recognition remains absent, is postponed, or breaks off—with serious consequences for one’s self-relation and societal participation.

This temporal structure is by no means self-evident but is historically and institutionally framed (Rosa, 2013; Torres, 2021). The accumulation of recognition over the life course—in the form of pension entitlements, professional experience, or social status—is the expression of a normative model of reciprocity: those who contribute receive something in return later. This notion of temporal reciprocity shapes many institutions of the welfare state. However, it presupposes certain conditions, such as continuity in employment biographies, life courses that can be planned, and the ability to postpone needs. Those who deviate from this norm—due to illness, caregiving responsibilities, dropping out of education, or precarious work—quickly find themselves in a recognition deficit that intensifies over time (Schweiger, 2024).

The theory of recognition can make these processes visible and subject them to critical analysis. It shows that social recognition is not only tied to the present but operates—or fails to operate—over time. Recognition is a social promise that must unfold over time. If it is not fulfilled, it turns into disappointment, resignation, or protest. A critique of societal orders of recognition must therefore always also address the temporality of these orders: Who receives what kind of recognition, and when? How long must one wait, perform, or endure before recognition occurs? Which groups are systematically excluded from building long-term relations of recognition? And what institutional changes would be necessary to correct these asymmetrical temporal relations? By expanding the theory of recognition to include an explicit temporal dimension, it gains in analytical precision and critical potential. It allows not only for determining whether recognition is granted or denied, but also for analyzing when, for how long, and under what conditions this happens. Especially in relation to phenomena such as poverty, precarity, or social insecurity, this perspective is central. For these states are not merely momentary deviations but are often the result of long-lasting, structurally embedded processes of non-recognition that unfold and solidify across entire life courses. A critical social theory that understands poverty and social inequality not merely as issues of distribution but as expressions of damaged relations of recognition must therefore also take their temporal dimension seriously.

## 3 Poverty, precarious work, and the life course as recognition and misrecognition over time

If recognition is understood as a fundamental social resource, then poverty is not merely an indicator of economic scarcity but a structural position within damaged relations of recognition. The theory of recognition makes it possible to grasp poverty not merely as a lack, but as a relational phenomenon systematically located within the social order of modernity (Schweiger, 2024; Schoneville, 2023). Precisely because modern societies are based on mutual recognition—because they promise self-realization, integration, and social esteem—poverty appears in these societies not only as economic disadvantage but as a violation of an implicit promise: namely, to be part of a community in which one is considered equal in value (Neuhäuser, 2016; Walker, 2019). At the same time, relations of recognition are never ahistorical or timeless, but embedded in temporal structures: who receives recognition, when, for how long, under what conditions, and with what entitlement, is not a neutral matter but an expression of normative orders. Therefore, poverty is not just a question of “how much” but of “how long,” of “when,” of “not yet” or “never.” The following section is dedicated to these temporal forms of poverty and develops a perspective in which poverty becomes visible as a chronic, biographically sedimented, and institutionally organized form of non-recognition and misrecognition.

### 3.1 Poverty as a temporally structured form of non-recognition and misrecognition

In modern welfare societies, poverty does not merely signify an insufficient supply of material goods (Townsend, 1979), but the systematic failure of societal relations of recognition. From this perspective, poverty is not simply a condition, but a structured social experience that accumulates, solidifies, and reproduces itself over time (Addison et al., 2009). Poverty is not only economically but also symbolically significant: it marks a position of lacking visibility, appreciation, and belonging—experienced both individually and institutionally. This becomes particularly evident when poverty is understood not as a momentary condition, but as a biographically embedded, processual configuration (Chamberlayne et al., 2002; Dewilde, 2003). The experiences associated with it are not only those of temporary scarcity, but of disappointed expectations, prolonged insecurity, shameful dependency, and a normatively disrupted life trajectory.

These experiences are not coincidental but follow a socially structured logic. In a society in which recognition is mediated through employment, consumption, and participation, poverty means exclusion from precisely these domains (Alcock, 2008; Byrne, 2005). Those who live permanently below what is considered a socially acceptable standard of living lose not only access to goods, but also to symbolic relevance. In a money-mediated society, the absence of money becomes the absence of status, the absence of social significance. Christoph Deutschmann has pointed out (Deutschmann, 2009) that money is the universal form of social integration in modern societies—those without money are excluded from the social world in a fundamental sense. In this way, poverty means not only limitation but de facto exclusion: from recognition, from participation, from self-efficacy. Particularly serious is the fact that poverty is not only imposed from the outside but inscribes itself into the self-relation of those affected. Those who live in poverty over the long term do not only feel disadvantaged but often feel they do not belong. Damaged self-relations emerge, shaped by shame, resignation, or even anger (Breitenbach et al., 2021; Jo, 2013; Ridge, 2011). The experience of not being seen, not being respected, not being needed undermines those basic forms of recognition on which a stable personal identity depends. Many accounts by people affected by poverty express precisely this: the feeling of being overlooked, forgotten, devalued. This perception is not merely subjective, but points to real exclusions—from work, education, public life, and political representation.

These theoretical considerations find concrete empirical support in studies documenting the institutional practices that systematically produce misrecognition among welfare recipients, particularly through what Turgeon et al. (2022) identify as “emotion rules” within welfare-to-work programs that require beneficiaries to perform gratitude, compliance, and optimism regardless of their actual circumstances or the adequacy of support provided. The research reveals how contact with social security systems frequently becomes a source of degradation rather than assistance, with recipients reporting experiences of surveillance, moral judgment, and conditional support that undermine rather than restore their social standing (Chassé et al., 2011). Jo (2013) analysis of the psycho-social dimensions of poverty demonstrates how these institutional encounters contribute to what she terms “shameful poverty”—a condition in which economic deprivation becomes intertwined with damaged self-relations that extend beyond material circumstances to encompass fundamental questions of social worth and belonging. The temporal dimension becomes particularly evident in how these experiences accumulate biographically, creating what Schmidt (2022) describes as ongoing challenges to human dignity that require constant “dignity work” by social workers to counteract the systematically degrading effects of means-testing, sanctioning, and moralizing practices embedded within contemporary welfare systems.

Moreover, poverty is in many cases not merely temporary but becomes effective as a biographical structure. Those who grow up in poverty (Attree, 2006; Terzi et al., 2023) have poorer educational opportunities, less access to healthcare, and a higher probability of remaining poor in adulthood. The experience of poverty, in this sense, is one that accumulates over time: what begins as a short-term bottleneck becomes, over the years, a normality, a constant condition, a biographical pattern. Poverty sediments itself in biographies, in life courses, in self-images. It is not merely a social situation, but a temporal structure—a form of “long-term suffering” that cannot be captured by numbers alone.

This persistence points to a central dimension of non-recognition and misrecognition that has thus far only been marginally addressed within the theory of recognition: its temporal logic. Misrecognition is not only violent injury, acute degradation, or targeted humiliation. Misrecognition can also consist in silent absence—in systematically “not being seen,” in “not being meant,” in “no longer being able to expect.” It is the constant knocking on doors that do not open, the long wait for attention, for participation, for a future. In this temporal form of misrecognition lies a double offense: on the one hand, the disappointment of the expectation to be recognized; on the other, the experience that this disappointment repeats itself, becomes normalized, and eventually turns into structure (Schweiger, 2020a).

In this sense, poverty is also an experience of temporal devaluation. For those who are poor, the present often appears as stagnation, as mere endurance. The future, by contrast, becomes unplannable, uncertain, threatening. The structure of reciprocity promised by many institutions of modern society—renouncing today to gain tomorrow—becomes a farce for people affected by poverty. Those who cannot invest cannot expect anything. Those who are not allowed or able to perform are given no prospect of return. The promise of the institutional life course—from education to work, from work to retirement—is interrupted, suspended, or never even activated (Dröge and Somm, 2005; Underlid, 2005). Poverty, in this sense, means not only having less, but not fitting into the temporal order of recognition.

Additionally, the institutional handling of poverty itself shapes recognition structures—and often fails to do so. Contact with social security systems is frequently described by those affected as degrading (Chassé et al., 2011; Turgeon et al., 2022; Schmidt, 2022; Stambe and Parsell, 2024). Aid is not granted as a right but as a favor. Support is not guaranteed but conditional: on availability, cooperation, and compliance. Those who rely on help are not addressed as equal citizens but as exceptions, as problem cases, as deviations. Precisely where the state intervenes to alleviate poverty, it often—unintentionally—reproduces its symbolic power. The practice of means-testing, the sanctioning of non-compliance, the moralizing of consumption habits or life choices are expressions of a paternalistic gaze that ties recognition to conditions many cannot—or will not—fulfill without losing their dignity.

This institutional practice of non-recognition or misrecognition is not merely a side effect, but part of a transformation in the rationality of social policy that can be observed in many countries (Feldman, 2019; Wacquant, 2010). The retreat of collective security systems in favor of activating measures, the primacy of individual responsibility, and the moral imperative of labor-market orientation have led to poverty no longer being addressed as a structural issue, but interpreted as individual failure. This intensifies the experience of shame, moral inferiority, and unworthiness. Those who are poor must explain, justify, improve themselves. The fact of being poor is no longer sufficient—one must prove that one does not deserve it, or that one will soon overcome it (Romano, 2017).

From this perspective, poverty becomes a litmus test of societal relations of recognition: where help is granted only with reservation, where participation becomes a reward for norm-conforming behavior, recognition is no longer understood as a right but as a certification of merit. Yet this undermines the core idea of modern justice—that people, by virtue of their dignity alone, are entitled to respect and participation. A society that administers poverty in such a way that it ties recognition to conditions that most poor people cannot fulfill fails not only in its social but in its moral promise. Poverty, as a temporal structure of damaged recognition, exemplifies that in modern societies the issue is not only one of just distribution, but of just temporal relations: Who is allowed to expect recognition, and when? Whose future counts? Whose present is visible? And whose past is recognized—or becomes the reason for continued exclusion? By expanding the theory of recognition to include a temporal perspective, it becomes possible to address these questions not only philosophically, but also through social theory and empirical analysis. In this sense, poverty is not merely a deviation, but an expression of a profound crisis in the temporal regimes of recognition.

### 3.2 Precarious work and the fragile life course

If poverty can be understood as exclusion from institutionalized relations of recognition, then precarious work marks an ambivalent position within those relations (Deranty, 2008; Standing, 2016; Castel, 2016): it does not represent complete exclusion, but rather an unstable, vulnerable, and fragile form of belonging. Precarious work is not characterized by a lack of employment, but by its insecurity, instability, and frequent devaluation. It concentrates a shift in societal recognition regimes—not through a withdrawal from labor as a central medium of social integration, but through its functional deregulation, normative degradation, and biographical unreliability. Those engaged in precarious work formally fulfill the societal demand for productivity, yet remain de facto in a zone of relative devaluation—between recognition and misrecognition, between inclusion and exclusion.

The concept of precarity does not only refer to an objective socioeconomic condition—such as temporary contracts, part-time work, agency work, or low wages—but also to an experience of social insecurity that deeply inscribes itself into everyday life (Millar, 2017; Deranty, 2008; Wimbauer and Motakef, 2020). Precarious work signals an erosion of the security, predictability, and social embeddedness of employment relations. Whereas the classical model of standard employment offered stability, status, and biographical orientation, precarious employment is marked by uncertainty, external control, and a lack of recognition. The normative expectation that work should not only be a means to an end, but a site of self-realization and societal respect, is increasingly disappointed under these conditions. This becomes especially evident in the relation between work and time. Precarious employment disrupts the temporal structure through which societal recognition is supposed to accumulate over the life course. The normative idea of the institutionalized life course—from education to employment to retirement, accompanied by growing autonomy, recognition, and security—is based on a specific temporal logic (Kohli, 2007; Dröge and Somm, 2005): those who invest today are meant to reap benefits tomorrow; those who perform should later be entitled to protection and care. In precarious conditions, this promise collapses. Those in unstable employment cannot develop long-term perspectives, build security, or constitute a stable identity through work. A kind of “counter-time” emerges—of insecurity, short-term adaptation, and permanent readiness—without the prospect of biographical progress.

The constant availability demanded of precarious workers points to a paradoxical form of devaluation: one is constantly active, productive, present—and yet remains economically and symbolically marginalized. The precarization of labor is not merely an expression of economic rationalization but part of a comprehensive transformation of social relations of recognition (Honneth, 2002). Work no longer automatically conveys social esteem (Voswinkel, 2012a). Rather, it is increasingly tied to conditions that many cannot fulfill—or do not wish to fulfill without sacrificing their dignity. The experience of working hard without receiving recognition has become a structural baseline for many in precarious conditions.

The existential dimension of precarious employment relationships becomes particularly evident in Millar (2017) analysis of how precarity operates as a structural condition that extends beyond immediate economic insecurity to encompass fundamental uncertainties about social position, future prospects, and the possibility of developing coherent life narratives. Standing (2016) comprehensive study of the precariat reveals how this “new dangerous class” experiences not merely job insecurity but what he terms “existential insecurity”—a condition characterized by the inability to develop stable expectations about work, income, or social recognition that extends across multiple life domains. The temporal aspects of this condition are further elaborated in Wimbauer and Motakef (2020) research on precariously employed individuals without partnerships, which demonstrates how employment instability intersects with relationship precarity to create cumulative recognition deficits that cannot be compensated through alternative social bonds. What emerges from these studies is a pattern whereby the flexibility demanded by contemporary labor markets—often celebrated as liberation from rigid employment structures—actually produces new forms of temporal discipline that require constant availability while providing no corresponding security or predictability, thereby undermining the very foundations upon which stable self-relations and social integration have traditionally been built.

What is at stake is not only a lack of status or low income. It is the erosion of the mutual promise that once underpinned work in industrial modernity (Holtgrewe et al., 2001): that it would provide not only effort but meaning, not only demands but support. In precarious conditions, this relationship shifts: work demands much—flexibility, resilience, constant availability—but gives little in return. It offers no future, no continuity, no recognition that extends beyond the moment. The result is a chronic experience of being unwanted, replaceable, “not being meant.” Recognition becomes a scarce resource, one that must be constantly fought for—without any guarantee of permanence.

The institutional effects of this dynamic are profound. In many cases, precarious employment prevents or severely impedes access to social security systems (Millar, 2017; Standing, 2016; Castel and Dörre, 2009). Those who cannot make continuous contributions, who only have interrupted or marginal employment records, are particularly vulnerable in old age, during illness, or in periods of unemployment. The idea of building social entitlements over a lifetime is undermined. What emerges is a kind of biographical fragmentation—not only of income, but of social belonging itself. Those affected live in a state of permanent transience—neither fully integrated nor clearly excluded, but always at risk.

Especially critical is the fact that precarious work undermines not only the present but also the future. It deprives workers not only of security in the here and now, but also of confidence in a safer, better future. It renders impossible the form of reciprocity that once characterized modern labor citizenship: those who contribute today should be protected tomorrow. In precarious life conditions, there is no guarantee that today’s effort will lead to recognition, security, or advancement in the future. The biographical perspective becomes a burden, a liability, a source of constant worry. Work loses its meaning as the structuring element of life and becomes a source of ongoing destabilization.

This dynamic has not only individual but structural consequences. The precarization of labor calls into question the entire recognition regime of the labor society. The idea that work is the central medium of social integration depends on the assumption that it is not only functionally but also normatively recognized. But when even full-time employment no longer provides social security, when achievements are no longer protected by rights but depend on discipline, conformity, and economic utility, work loses its legitimating force. Society then produces not only poverty despite work, but alienation through work—an alienation not caused by a lack of motivation, but by a lack of recognition.

From this perspective, precarity appears not as a transitional phenomenon, but as a new social normal in which life courses are no longer linear but fragmented, in which belonging is no longer granted permanently but conditionally (Beck and Beck-Gernsheim, 2002). Precarious work is not an individual exception but an expression of structural insecurity that increasingly affects all areas of social life. It represents a profound erosion of the temporal regimes through which recognition is mediated. The experience that one’s own time cannot be “invested”—because there is no stable expectation of return—destroys the very sense of social participation.

This experience also leaves marks at the level of social subjectivity. Those who live in precarious conditions over the long term not only lose trust in institutions but also in themselves. The constant flexibilization leads to deep insecurity in one’s identity, to a feeling of interchangeability, powerlessness, and invisibility (Herzog, 2018; Gaisbauer and Kapferer, 2016). The desire for recognition remains—but it goes unfulfilled. Instead, a kind of chronic absence of expectation arises, a mode of survival without perspective. In this constellation, precarity is not merely economic but existential: it undermines the foundation upon which both a stable relationship to the self and the hope for social belonging rest.

This makes clear: precarious work is not a marginal deficit but a central form of modern recognition insecurity. It reveals that the promise of performance and reward, of effort and recognition, of investing time and gaining time has become fragile. The precarization of the life course exemplifies the transformation of societal relations of recognition—and points to a deep crisis in the temporal regime that once gave those relations meaning and direction.

## 4 Social critique of temporal regimes of recognition

Modern societies structure recognition not only through content or performance, but to a significant extent through time. Recognition occurs not merely because something is said, done, or achieved, but because it happens at the right time, lasts long enough, or can be expected during the appropriate life stage. From this perspective, it is not only the social conditions of recognition that matter, but also its temporal framing: those who arrive too late, act too early, hesitate too long, or do not perform in time may be denied recognition just as much as those whose contributions appear non-normative or insufficiently continuous. The concept of “temporal regime” refers precisely to those societal orders that impose normative expectations on the temporal structure of life—on the rhythm, pace, sequence, and duration of actions, developments, and performances. Recognition thus becomes not only a social but a temporal phenomenon.

Temporal regimes organize the social by determining when what is supposed to happen (Torres, 2021)—when one should learn, work, reproduce, consume, relax, or retire. They generate notions of normality by which biographies are measured and evaluated. Those who live in sync with these rhythms are seen as adapted, predictable, reliable—and are granted corresponding recognition. Those who fall out of step, move too fast, lag behind, or remain stuck are pushed to the margins of the symbolic order. Time, then, is not merely a neutral medium of social action but a normative structure through which belonging, value, and respect are distributed. Yet it is precisely this normativity that also harbors its dark side: those who fall out of rhythm—due to illness, migration, caregiving, poverty, educational dropout, or unemployment—are often denied not only support but also recognition. Temporal deviations are interpreted as moral or social deficits. Someone who is unemployed “too long” is seen as unwilling; someone who enters professional life “too late,” as a failure; someone who retires “too early,” as a burden. Life time becomes a resource that must be optimized, measured, and justified—a process that subordinates time itself to normative ordering.

This development is exacerbated under conditions of neoliberal transformation. The classical temporal regime of the welfare state—with stable transitions, secured life phases, and plannable biographies—is increasingly being replaced by a fragmented, individualized, and flexibilized model (Hammershøj, 2009; Beck and Beck-Gernsheim, 2002; Masquelier, 2017). Life courses are no longer meant to be standardized but “dynamic”; employment paths no longer stable but “mobile”; educational trajectories no longer linear but “lifelong.” What initially sounds like liberation proves, upon closer inspection, to be a new form of normativity: the temporal expectations do not disappear; they merely become more fluid—and therefore harder to contest, yet no less powerful. The result is a new recognition regime in which temporal adaptability becomes a prerequisite for visibility, participation, and status. Those who manage to conform to this accelerated, unbounded temporal regime are at least in principle eligible for societal recognition. But this “principle” is deceptive: in reality, the ability to be flexible, mobile, and permanently available is unevenly distributed. It depends on resources such as health, education, social background, gender, caregiving responsibilities, or migration status. What appears to be a space of freedom is in fact a new grid by which biographies are measured—a grid that many are unable to meet.

This becomes particularly evident in the realm of employment, which continues to represent the central medium of societal recognition (Honneth, 2010). Those who work are regarded as productive, useful, responsible—and receive not only income, but also respect, visibility, and social status (Voswinkel, 2012b). Yet this recognition is no longer bound to the mere fact of working, but to its temporal structure: it should be continuous, available, efficient, and plannable. Those who are only briefly employed, who work part-time, who show gaps in their employment histories, or who perform care work (Wimbauer, 2023; Gregoratto, 2016), often fall outside the framework of recognition. It is precisely the temporal dimension that here becomes a mechanism of exclusion: not only what counts, but when, for how long, and how often.

The normative violence of temporal recognition regimes becomes particularly evident in empirical research on gender and care work, where studies consistently document how women—especially those with caregiving responsibilities—face systematically irreconcilable temporal demands that prevent them from achieving recognition in any single domain (Gregoratto, 2016). Hübgen (2018) comparative analysis of lone mothers’ poverty risks across European contexts reveals how institutional arrangements fail to acknowledge the temporal complexity of combining care work with paid employment, resulting in what she terms being “only a husband away from poverty”—a condition that exposes the gendered assumptions embedded within supposedly neutral welfare institutions. The temporal dimension becomes particularly salient in these analyses, as they reveal how contemporary recognition regimes demand temporal flexibility from individuals while maintaining institutional rigidities that penalize those unable to conform to standardized life course expectations. Research by Attree (2006) on the social costs of child poverty further demonstrates how these temporal pressures reproduce intergenerationally, as children in poor families experience not only immediate material deprivation but also restricted access to the temporal resources—such as time for education, play, and development—that are prerequisites for future social mobility and recognition, thereby perpetuating cycles of temporal disadvantage that extend across generations.

This dynamic affects not only individual recognition but also institutional structures. Social security systems based on the classical life course model come under pressure when biographies no longer follow the expected trajectory. Those who, due to caregiving, illness, or unstable employment, are unable to build sufficient pension entitlements are not only materially at risk but also symbolically devalued—as people who have “not lived properly,” “not contributed enough,” or “not acted with foresight.” The temporal logic of institutions remains normative, even though the reality of life courses has long changed. Here, a structural tension between recognition and time becomes apparent: while the societal ideal of a successful life still relies on temporal linearity, planning, and reciprocity, precarious life realities systematically undermine this ideal. Those who do not move along the expected temporal axes are denied not only support but also legitimacy. The result is a creeping pathologization of deviation, which is not understood as an expression of structural inequality but as individual failure. In this process, social hierarchies are reproduced not only through resources but through time: through the right to plan, to endure, to have a future.

This connection between time and recognition is also particularly relevant at the level of subjective experience. Many people in precarious life situations report a loss of temporal orientation: the future appears unplannable, the present burdensome, the past a failure. Time is no longer experienced as a continuum of development, but as a series of crises, transitions, ruptures. This subjective perception of time stands in stark contrast to the societal ideal of an orderly, progressive, meaningful life course—and thus reinforces the experience of non-belonging. Those who are unable to structure their time meaningfully, because their circumstances do not allow it, become marginalized not only materially but also symbolically.

Against this backdrop, it becomes clear that time is not only a precondition for recognition, but a contested field of its political and social organization (Goodin, 2010; Tyssedal, 2021). Temporal justice becomes a prerequisite for participation, respect, and equality. But this justice is seriously at risk when time itself becomes an exclusive resource—through temporary employment contracts, through the overload of reconciling work and family, through the pressure of lifelong upskilling, through constant expectation management in uncertain conditions. Time becomes a commodity, a performance, a debt—and thus a boundary of recognition. A critical theory of recognition must therefore address the question of what temporal orders prevail in a society, whom they serve, and whom they exclude. It must analyze how normative temporal ideas—of efficiency, linearity, life phases, or reciprocity—contribute to the reproduction of social inequality. And it must envision alternative models of time: ones in which life is not evaluated according to its speed or planability, but according to its diversity, openness, and unconditionality.

It is important to stress: not every form of temporal structuring is problematic. Time needs order, orientation, rhythm. It becomes problematic where this order is normatively overdetermined—where it demands conformity rather than enabling diversity; where it disciplines rather than protects; where it legitimizes exclusion rather than fostering participation. A society that ties recognition to time norms without distributing the preconditions for meeting them fairly fails the promise of democratic equality. In this sense, the critique of temporal regimes of recognition is not a niche concern, but central to understanding modern inequality. It reveals that exclusion is not caused solely by property, education, or origin, but also by the unequal distribution of time and by the normative charging of biographical rhythms. A more just society would not only be one with less poverty, but one in which people have equal chances for recognition—regardless of their control over time, their life stage, or their biography.

## 5 Conclusion and outlook

This paper has sought to expand the theory of recognition through a systematic reflection on its temporal dimension and to apply it to the analysis of poverty, precarious work, and biographical life courses. At the center stood the thesis that recognition is not only socially but also temporally structured, and that many forms of social exclusion—particularly poverty and precarity—can be understood as ruptures, blockages, or denials within temporally organized relations of recognition. Recognition was thus employed not merely as a moral-philosophical concept, but as a social-theoretical tool to analyze the logic of modern inequality.

It was first shown that recognition in modern societies does not occur solely in interpersonal relationships but is fundamentally mediated by institutional structures. These institutions—particularly the education system, labor market, and welfare state—structure recognition through time: who is considered worthy of recognition and when, who can expect what in return, and in which life phase certain forms of esteem are granted or withheld, all follow a historically evolved temporal regime. This regime is based on the idea of a “normal” life course, divided into phases of preparation, productivity, and withdrawal—and deviation from this model is typically marked as deficiency or failure. Through the analysis of poverty, it became clear how this temporally structured recognition can systematically fail. Poverty was reconstructed not only as a state of lack, but as a processual experience of non-recognition and misrecognition. It manifests as disappointed expectations, biographically accumulated exclusion, institutional humiliation, and the inability to experience oneself as a full member of society within the dominant temporal regime. Particularly troubling is that poverty is not marked by isolated episodes of exclusion, but by a structural decoupling from reciprocity, planning, and recognition—central dimensions of the social time regime. In this perspective, poverty also means: being unable to live in sync with the societal rhythm.

Precarious work, on the other hand, marks a different form of damaged temporal relations. It is not complete exclusion but a fragile form of belonging that is constantly contingent on proving oneself. People in precarious employment often work continuously, yet receive little recognition because their working time is not coded as permanent, reliable, or valuable. The temporal structure of work—through fixed-term contracts, agency work, project-based employment, or permanent availability—undermines the ability to build a stable relation to the self or to expect social security over time. Classical models of life planning thus lose their validity and are replaced by a mode of permanent uncertainty, which has not only economic but existential effects. The fourth section made clear that these changes are not isolated phenomena but point to a broader problem within temporally organized recognition regimes. In modern societies, time is not distributed neutrally but normatively structured—and serves as a silent medium of social selection. Those who cannot conform to the dominant temporal regime—due to structural, biographical, or social reasons—not only lose access to material resources, but also to symbolic significance. The result is a social order in which time itself becomes the boundary of recognition.

This insight allows for a critical redefinition of what social participation means. It is not enough to guarantee rights or provide material transfers if the recognition that should accompany these rights remains conditional on adhering to normed life paths. A just society must therefore be measured not only by its systems of distribution, but also by the quality and openness of its temporal structures. The question is not just: Who has how much? But also: Who is allowed to expect something, and when? Who is entitled to claim recognition—and who remains indefinitely in a state of waiting, exclusion, or invisibility? Looking forward, the challenge lies in deepening this analysis both empirically and normatively. Empirically, research should investigate how specific institutions—such as job centers, pension funds, or educational institutions—actually mediate or withhold recognition in temporal terms. Biographical studies could also show how people themselves experience their life time within the tension between recognition and misrecognition. Normatively, the question must be asked what a different temporal regime could look like: one not based on linear productivity, but on continuity of belonging; one that does not tie promises of the future to labor power, but envisions the present as a site of unconditional recognition.
